# Effectiveness of predicting in-hospital mortality in critically ill children by assessing blood lactate levels at admission

**DOI:** 10.1186/1471-2431-14-83

**Published:** 2014-03-28

**Authors:** Zhenjiang Bai, Xueping Zhu, Mengxia Li, Jun Hua, Ying Li, Jian Pan, Jian Wang, Yanhong Li

**Affiliations:** 1 Pediatric Intensive Care Unit, Children’s Hospital affiliated to Soochow University, Suzhou, China; 2 Department of Neonatology, Children’s Hospital affiliated to Soochow University, Suzhou, China; 3 Department of Nephrology, Children’s Hospital affiliated to Soochow University, Suzhou, China; 4 Institute of Pediatric Research, Children’s Hospital affiliated to Soochow University, Suzhou, China

**Keywords:** Blood lactate, Critically ill children, Cut-off value, In-hospital mortality, Pediatric risk of mortality III (PRISM III), Predictive test

## Abstract

**Background:**

Hyperlactatemia upon admission is a documented risk factor for mortality in critically ill adult patients. However, the predictive significance of a single lactate measurement at admission for mortality in the general population of critically ill children remains uncertain. This study evaluated the predictive value of blood lactate levels at admission and determined the cut-off values for predicting in-hospital mortality in the critically ill pediatric population.

**Methods:**

We enrolled 1109 critically ill children who were admitted to a pediatric intensive care unit between July 2008 and December 2010. Arterial blood samples were collected in the first 2 hours after admission, and the lactate levels were determined. The Pediatric Risk of Mortality III (PRISM III) scores were calculated during the first 24 hours after admission.

**Results:**

Of the 1109 children admitted, 115 (10.4%) died in the hospital. The median (interquartile range) blood lactate level in critically ill children was 3.2 mmol/l (2.2-4.8). Among the children, 859 (77.5%) had a lactate concentration >2.0 mmol/l. The blood lactate level upon admission was significantly associated with mortality (odds ratio [OR] = 1.38; 95% confidence interval [CI], 1.30-1.46; *p* <0.001), even after adjustment for age, gender, and illness severity assessed by PRISM III (OR = 1.27; *p* <0.001). Multivariate regression analysis showed that a high blood lactate level (OR = 1.17; 95% CI, 1.07-1.29; *p* = 0.001), a high PRISM III score (OR = 1.15; 95% CI, 1.11-1.20; *p* <0.001), and a low serum albumin (OR =0.92; 95% CI, 0.88-0.96; *p* <0.001) were independent risk factors for mortality in critically ill children. Blood lactate achieved an area under-the-receiver-operating-characteristic curve (AUC) of 0.79 (*p* <0.001) for predicting mortality that was similar to that of PRISM III (AUC = 0.82; *p* <0.001). The *p-*value for a comparison of both AUCs was 0.318. Blood lactate displayed a sensitivity of 61% and a specificity of 86% in predicting mortality at the optimal cut-off value of 5.55 mmol/l, and the positive and negative likelihood ratios were 4.5 and 0.45, respectively.

**Conclusions:**

A high blood lactate level at admission is independently associated with and predictive of in-hospital mortality in the general population of critically ill children.

## Background

Early recognition of children who are at high risk for mortality allows for timely changes in therapy and improves the overall outcomes. Although critical care in pediatric patients has changed dramatically over the last several decades, there has been no consistent marker for obtaining predictions of mortality in a general population of critically ill children.

Lactate has been used as a marker of tissue hypoperfusion and cellular hypoxia, and hyperlactatemia is significantly associated with mortality [1-5]. Previous studies evaluated the predictive value for mortality of both a single lactate screening measurement at admission and serial lactate measurements [1,6]. Hyperlactatemia in adult patients upon admission to a general medical intensive care unit was a predictive marker identifying patients who were at high risk for death [7].

Despite the publication of a few studies that investigated the association of admission hyperlactatemia with mortality in the pediatric population [6,8-16], information on its importance in a general population of critically ill children is limited [9,14,15]. The predictive value of a single lactate level assessed at admission, unlike serial measurements of lactate, is controversial in the general population of critically ill children [9,14,15]. One study of 75 patients admitted to the pediatric intensive care unit (PICU) found that blood lactate levels assessed at 24 hours after admission, but not at 6 hours, had better sensitivity or specificity as a predictor of death [15]. Persistent hyperlactatemia 24 hours after PICU admission is associated with mortality, as shown in a study of 50 patients with early hyperlactatemia (lactate >2 mmol/l within 6 hours of admission). In that study group, however, the lactate level measured at admission did not differ significantly between survivors and non-survivors [9]. In contrast to these reports, a recently published clinical study conducted in a retrospective cohort suggested that the blood lactate concentration at PICU admission predicts mortality independent of the pediatric index of mortality (PIM) [14].

The predictive significance for a single lactate measurement as a screening method for children upon admission to the PICU remains uncertain. Furthermore, a lactate level that consistently predicts mortality in the general population of critically ill children has not been identified. We hypothesized that the blood lactate level upon admission to PICU is significantly associated with mortality in critically ill children. This study evaluated the predictive value of admission blood lactate and determined the cut-off values for predicting in-hospital mortality in the general population of critically ill children.

## Methods

All children admitted to the PICU from July 2008 to December 2010 were considered for inclusion in the study. The criteria for PICU admission were adopted from guidelines for developing admission and discharge policies for the pediatric intensive care unit [17]. The study exclusion criteria were death in the first 2 hours after admission and unexpectedly discharged or transferred to another hospital. The Institutional Review Board of the Children’s Hospital of Soochow University approved the study. Informed consent was obtained from the parents of the children enrolled in the study.

### Clinical and laboratory data collection

Clinical and laboratory data were collected on the day of admission and included age, gender, admission diagnosis, illness severity assessed by the Pediatric Risk of Mortality III (PRISM III) score, routine hematological tests, a serum biochemical profile, and arterial blood gas analysis. Clinical status, comorbidities, therapeutic interventions, and medication were recorded daily until hospital discharge or death.

The presence of multi-organ dysfunction syndrome (MODS) during hospitalization was determined according to the criteria modified from the international pediatric sepsis consensus conference [18]. The maximum number of dysfunctional organs was 6 (cardiovascular, respiratory, neurologic, hematologic, renal, and hepatic). Children with dysfunction in 3 or more organs were compared to those with fewer than 3 dysfunctional organs.

### The PRISM III score

The PRISM III score was calculated to assess the illness severity according to methods described in the original studies [19,20]. In brief, PRISM III was scored based on age-related physiological parameters collected during the first 24 hours after admission, including systolic blood pressure, heart rate, temperature, pupillary reflexes, mental status, acidosis (pH and total CO2), pCO2, pO2, glucose, potassium, creatinine, blood urea, white blood cell count, platelet count, and prothrombin or partial thromboplastin time.

### Clinical outcome

In-hospital mortality was defined as a death occurring in the hospital after PICU admission. A favorable outcome was defined as a child who was discharged to home directly from the PICU or after transfer to another department in our hospital.

### Blood sample collection and measurement

To determine the blood lactate level at admission, an arterial sample was collected in the first 2 hours after a child’s admission to the PICU. The sample was collected directly into a tube containing anticoagulant. The blood concentration of lactate was measured immediately using an automatic biomedical blood gas analyzer (CCX, NOVA Biomedical, Waltham, MA, USA) as part of a routine panel of blood gas tests in our clinical laboratory with the electrode method, and it was expressed in millimole per liter (mmol/l). The blood gas analyzer was calibrated every 2 hours and checked twice per day. The detection limit for lactate was 0.5 mmol/l. The coefficient of variation was 7.5% at the low level and 5.0% at the high level. The normal range in children is 0.5-2.5 mmol/l. The laboratory investigators were blinded to the sample sources and clinical outcomes.

### Statistical analysis

Statistical analyses were done by using SPSS 13.0. Assumptions of normality and homogeneity of variance were first checked. For continuous variables with a skewed distribution, descriptive results were expressed as medians and interquartile ranges. The Mann–Whitney *U* test was used to determine the differences between two groups, and the Kruskal-Wallis H test was used to analyze the differences among groups. Univariate binary and multivariate logistic regression analyses were performed to investigate whether blood lactate was independently associated with in-hospital mortality. The model fit was assessed with the Hosmer–Lemeshow goodness-of-fit test. A non-significant value for the Hosmer-Lemeshow Chi-square test suggests an absence of biased fit. Analysis of the area under the curve (AUC) of the Receiver Operating Characteristic (ROC) curve was constructed to assess the predictive strength. The nonparametric method of Delong was used to compare significant difference between AUCs (Sigmaplot 10.0 software). Sensitivity, specificity, and positive and negative likelihood ratios and predictive values were calculated at different cut-off values. Optimal cut-off points to maximize both sensitivity and specificity were also determined. All probability values are two-sided. Differences with *p* values <0.05 were considered to be statistically significant.

## Results

### Patient characteristics

The study enrolled 1109 critically ill children, including 1045 children with a medical admission diagnosis and 64 with a surgical admission diagnosis. Of the total 1204 children admitted to the PICU during the study period, 54 were first excluded: 37 because of refusal to participate by parents and 17 due to failure to obtain an arterial blood sample during the first 2 hours after admission. Of the 1150 children who had lactate values from arterial blood samples, 41 were excluded: 2 died in the first 2 hours after admission, 3 were transferred to another hospital, and 36 were unexpectedly discharged due to economic reasons. Major medical admission diagnoses included respiratory diseases (39.9%), neurological diseases (24.3%), gastrointestinal diseases (7.3%), cardiovascular diseases (5.1%), sepsis (4.0%), hematologic/oncologic diseases (3.7%), poisoning (2.9%), and others (7.0%). There was no significant difference between the included and excluded children with regard to age (median [interquartile range]: 1.00 [0.25-3.00] *vs* 0.67 [0.25-3.00] years, *p* = 0.221), gender (male/female: 689/420 *vs* 23/18, *p* = 0.513), or PRISM III score (3 [2-5] *vs* 3 [0–7], *p* = 0.740).

Of the total 1109 children, 115 (10.4%) died in the hospital. The median time from PICU admission to death was 48 hours (min-max range: [2.5-1176]; interquartile range [24–84]) after admission. Children who were discharged to home were considered to have a favorable outcome. The demographic and clinical characteristics and laboratory findings on the day of admission are compared between survivors and non-survivors in Table 1. The concentration of blood lactate at admission in surviving children was significantly higher than in those who did not survive (p <0.001).

**Table 1:** Comparison of demographic and clinical characteristics and laboratory findings on admission day between survival and non-survival critically ill children

**Characteristics**	**Survivors (n =994)**	**Non-survivors (n =115)**	***P *** **value**
Age, years	1.00 [0.29-3.00]	1.00 [0.25-3.13]	0.680
Gender, male/female	629/365	60/55	0.025
PRISM III score	3 [0–6]	12 [5–23]	<0.001
MODS ≥3^a^, n	63 (6.3)	58 (50.4)	<0.001
Need for mechanical ventilation^b^, n	172 (17.3)	67 (58.3)	<0.001
Laboratory findings on admission day^c^			
Blood lactate, mmol/l	3.10 [2.10-4.50]	6.60 [3.8-11.95]	<0.001
Serum albumin, g/l	42.6 [39.6-45.8]	36.5 [30.2-42.0]	<0.001
Blood glucose, mmol/l	7.12 [5.71-9.75]	10.10 [5.73-21.29]	<0.001
Serum creatinine, μmol/l	27.9 [23.20-36.78]	42.7 [27.45-67.65]	<0.001
Blood urea nitrogen, μmol/l	3.71 [2.61-5.08]	4.90 [3.69-6.71]	<0.001
Serum total bilirubin, μmol/l	6.48 [4.26-9.94]	8.03 [4.20-13.96]	0.023
Blood bicarbonate, mmol/l	20.8 [18.0-23.9]	17.3 [12.0-21.1]	<0.001
Arterial pH	7.415 [7.349-7.472]	7.359 [7.181-7.438]	<0.001

Values are median [interquartile range]. Numbers in parentheses denote percentages.

*MODS*, multi-organ dysfunction syndrome; *PRISM III,* pediatric risk of mortality III.

^a^MODS developed during hospitalization. ^b^Administration during hospitalization. ^c^The first available laboratory values during the first 24 hours after admission.

### Comparison of data in children with different concentrations of blood lactate

Blood lactate was detectable with a range of 0.6-28.3 mmol/l in 1101 samples. For undetectable levels, the values of blood lactate were arbitrarily given values of 0.4. The median blood lactate level measured in the first 2 hours after admission in critically ill children was 3.2 mmol/l. Among the children, 859 (77.5%) had a lactate concentration >2.0 mmol/l, including 485 (43.7%), 204 (18.4%), 79 (7.1%), 34 (3.1%) and 57 (5.1%) who had lactate concentrations of 2.1-4.0, 4.1-6.0, 6.1-8.0, 8.1-10.0 and greater than 10.0 mmol/l, respectively.

A comparison of the demographic and clinical characteristics and the laboratory findings collected on the day of admission among children is shown in Table 2. The incidence of mortality in critically ill children was significantly associated with increased blood lactate levels (p <0.001). A significant increase in the PRISM III scores (p <0.001) and the blood glucose concentrations (p <0.001) was associated with increases in the blood lactate levels. In contrast, the blood bicarbonate concentrations (p <0.001) and arterial pH values (p <0.001) were significantly decreased with increases in the blood lactate levels.

**Table 2:** Comparison of demographic and clinical characteristics and laboratory findings on admission day among children with different concentrations of blood lactate

**Admission blood lactate, mmom/l**	**0.0-2.0**	**2.1-4.0**	**4.1-6.0**	**6.1-8.0**	**8.1-10.0**	**>10.0**	***P *** **value**
n	250	485	204	79	34	57	
Age, years	1.75 [0.58-4.00]	1.00 [0.33-3.00]	0.67 [0.25-2.00]	0.54 [0.25-3.50]	0.42 [0.17-1.25]	0.92 [0.33-3.00]	<0.001
Gender, male/female	163/87	304/181	122/82	60/19	9/25	31/26	<0.001
PRISM III score	3 [0–6]	3 [0–6]	3 [0.75-7]	5 [2–11]	8 [4–15.75]	14 [6–24]	<0.001
MODS ≥3^a^, n	11 (4.4)	24 (4.9)	20 (9.8)	20 (25.3)	12 (35.3)	34 (59.6)	<0.001
Mechanical ventilation^b^, n	29 (11.6)	95 (19.6)	48 (23.5)	23 (29.1)	13 (38.2)	31 (54.4)	<0.001
In-hospital mortality, n	7 (2.8)	27 (5.6)	18 (8.8)	14 (17.7)	9 (26.5)	40 (70.2)	<0.001
Laboratory findings on admission day^c^							
Albumin, g/l	42.6	42.7	42.0	40.2	39.6	32.8	<0.001
	[39.1-45.8]	[39.8-46.0]	[39.9-45.7]	[34.2-44.5]	[35.9-43.15]	[29.0-40.0]	
Glucose, mmol/l	6.50	6.90	7.60	8.90	11.35	16.27	<0.001
	[5.35-8.00]	[5.70-9.00]	[6.15-11.37]	[6.65-14.02]	[7.40-21.21]	[10.78-27.90]	
Creatinine, μmol/l	28.2	27.4	27.8	31.7	37.6	62.4	<0.001
	[23.2-35.5]	[23.2-35.0]	[23.1-37.5]	[22.8-48.8]	[25.9-60.1]	[43.0-79.7]	
BUN, μmol/l	3.64	3.70	3.59	4.46	4.87	4.72	<0.001
	[2.62-5.04]	[2.62-4.92]	[2.35-5.12]	[2.85-6.69]	[3.04-6.48]	[3.80-6.92]	
Total bilirubin, μmol/l	6.08	6.50	7.01	9.43	4.91	6.71	<0.001
	[4.20-8.50]	[4.21-9.73]	[4.73-11.69]	[6.18-17.28]	[3.33-8.89]	[4.03-12.61]	
Bicarbonate, μmol/l	21.4	20.8	20.8	17.7	15.9	11.5	<0.001
	[18.8-24.4]	[18.4-24.0]	[17.9-23.8]	[14.8-21.4]	[11.7-20.4]	[6.6-15.3]	
Arterial pH	7.439	7.421	7.390	7.368	7.267	7.177	<0.001
	[7.385-7.488]	[7.364-7.476]	[7.332-7.456]	[7.304-7.464]	[7.141-7.338]	[7.005-7.272]	

Values are median [interquartile range]. Numbers in parentheses denote percentages.

*BUN*, blood urea nitrogen*; MODS*, multi-organ dysfunction syndrome; *PRISM III,* pediatric risk of mortality III.

^a^MODS developed during hospitalization. ^b^Administration during hospitalization. ^c^The first available laboratory values during the first 24 hours after admission.

### Association of blood lactate level with in-hospital mortality

Univariate binary and multivariate logistic regression analyses were performed to investigate whether the blood lactate level at admission was independently associated with in-hospital mortality (Table 3). Age, gender, illness severity as assessed by the PRISM III score, and laboratory findings collected on the day of admission that were potentially associated with in-hospital mortality were included in the analyses. The following factors were significantly associated with in-hospital mortality in the unadjusted binary logistic regression analysis: gender, PRISM III score, blood lactate level, albumin, glucose, creatinine, urea nitrogen and bicarbonate, and arterial pH value. The odds for in-hospital mortality increased by 38%, for every 1 mmol/l increase in blood lactate (OR = 1.38; 95% CI, 1.30-1.46; *p* <0.001). The association of blood lactate levels with in-hospital mortality remained significant after adjusting for age, gender, and the severity of illness as assessed by the PRISM III score (OR = 1.27; 95% CI, 1.19-1.35; *p* <0.001).

**Table 3:** Univariate and multivariate logistic regression analyses of variables potentially associated with in-hospital mortality

	**Univariate binary logistic regression**		**Multivariate logistic regression**	
	**OR (95**% **CI)**	***P *** **value**	**OR (95**% **CI)**	***P *** **value**
Age	1.02 (0.96-1.08)	0.542	1.06 (0.96-1.16)	0.249
Gender	1.58 (1.07-2.33)	0.021	1.11 (0.63-1.97)	0.715
PRISM III score	1.18 (1.15-1.21)	<0.001	1.15 (1.11-1.20)	<0.001^e^
Laboratory findings on admission day^f^				
Lactate	1.38 (1.30-1.46)	<0.001^a^	1.17 (1.07-1.29)	0.001^b,c^
Albumin	0.86 (0.83-0.89)	<0.001	0.92 (0.88-0.96)	<0.001^d^
Glucose	1.11 (1.08-1.13)	<0.001	1.00 (0.96-1.03)	0.829
Creatinine	1.01 (1.00-1.01)	<0.001	0.99 (0.98-1.01)	0.428
Urea nitrogen	1.05 (1.01-1.08)	0.006	1.03 (0.94-1.12)	0.556
Total bilirubin	1.01 (1.00-1.01)	0.010	1.01 (0.99-1.01)	0.080
Bicarbonate	0.89 (0.87-0.93)	<0.001	1.03 (0.98-1.08)	0.217
Arterial pH	0.01 (0.00-0.03)	<0.001	0.54 (0.06-4.75)	0.580

*CI*, confidence interval; *OR*, odds ratio; *PRISM III,* pediatric risk of mortality III.

The *p* value of the Hosmer-Lemeshow goodness-of-fit test for the multivariate logistic regression model was 0.611.

^a^The association of blood lactate with in-hospital mortality remained significant after adjustment for age, gender, and the PRISM III score (OR = 1.27; 95% CI, 1.19-1.35; *p* <0.001). ^b^The association of the blood lactate level with in-hospital mortality remained significant after adjustment for serum albumin and the PRISM score (OR = 1.17; 95% CI, 1.09-1.26; *p* <0.001). ^c^Odds ratio represents the increase in risk per 1 mmom/l increase in lactate concentration. ^d^Odds ratio per 1 g/l increase in albumin level. ^e^Odds ratio per 1-point increase in PRISM III score. ^f^The first available laboratory values during the first 24 hours after admission.

Multivariate logistic regression analysis identified blood lactate (OR = 1.17; 95% CI, 1.07-1.29; *p* = 0.001), serum albumin (OR per 1 g/l increase = 0.92; 95% CI, 0.88-0.96; *p* <0.001), and the PRISM III score (OR per 1-point increase = 1.15; 95% CI, 1.11-1.20; *p* <0.001) as independent factors that were significantly associated with in-hospital mortality in critically ill children. The Hosmer-Lemeshow goodness-of-fit test for the multivariate logistic regression model was not significant (*p* = 0.611), indicating that the model adequately fits the data. Furthermore, the association between blood lactate levels and in-hospital mortality remained significant after adjusting for the PRISM III score and serum albumin (OR = 1.17; 95% CI, 1.09-1.26; *p* <0.001).

### Ability of blood lactate level to predict in-hospital mortality

**Figure 1:**
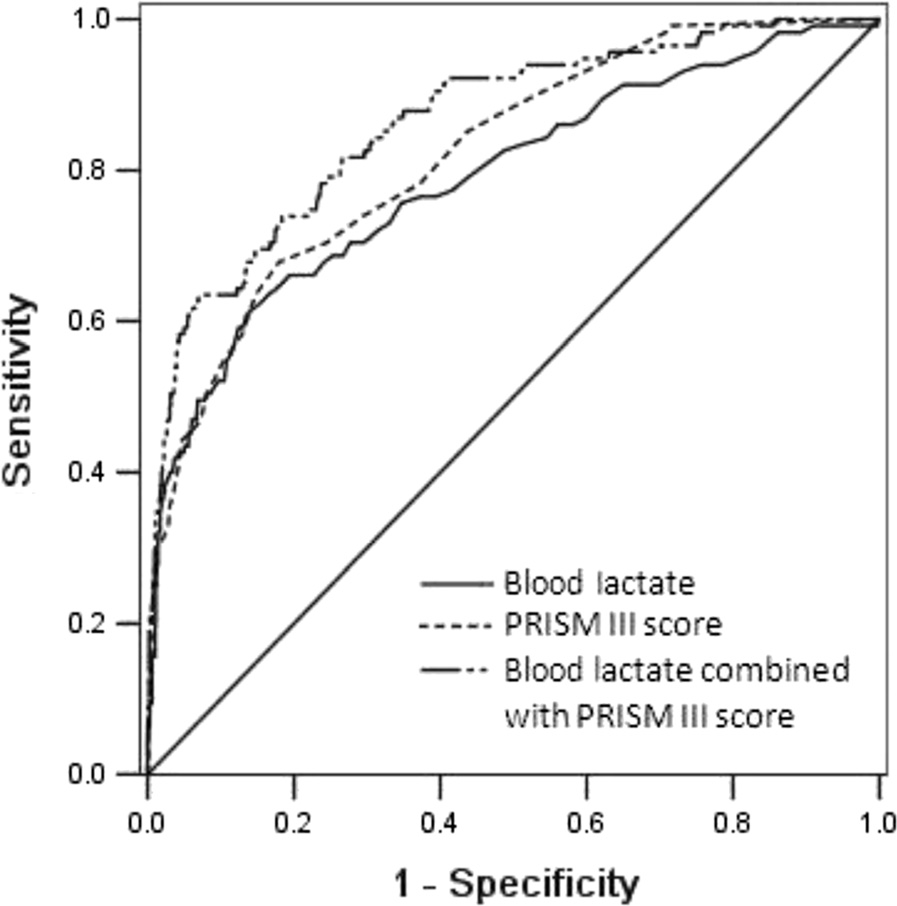
**Receiver operating characteristic curves for the ability of blood lactate, PRISM III, and blood lactate combined with PRISM III to predict in-hospital mortality in generally critically ill children (n = 1109).** The area under the receiver operating characteristic curve for blood lactate, PRISM III, and blood lactate combined with PRISM III were 0.79, 0.82 and 0.86, respectively, with a Hosmer-Lemeshow goodness-of-fit *p* value >0.05.

The predictive ability of blood lactate levels from all children admitted to the PICU (n = 1109) for in-hospital mortality (n = 115) was assessed (Table 4). The level of blood lactate was predictive of in-hospital mortality and achieved AUC of 0.79 (95% CI, 0.74-0.84; *p* <0.001). This AUC is similar to the PRISM III score (AUC = 0.82; 95% CI, 0.78-0.86; *p* <0.001) for predicting the in-hospital mortality. The *p-*value for comparison of both AUCs was 0.318. Combining blood lactate levels with the PRISM III score improved the predictive performance (AUC = 0.86; 95% CI 0.83-0.90; p <0.001), which is better than blood lactate alone (p = 0.018), but not significantly better than PRISM alone (p = 0.135). Figure 1 shows the ROC curves and the AUC of the blood lactate level at admission, the PRISM III score, and the combination of the blood lactate with the PRISM III score for predicting the in-hospital mortality of critically ill children.

**Table 4:** Predicting performance of admission blood lactate and PRISM III for in-hospital mortality

	**AUC**	**95% CI**	***P *** **value**	**Optimal cut-off value**	**Sensitivity**	**Specificity**
Admission blood lactate	0.79	0.74-0.84	<0.001	5.55 mmol/l	61%	86%
PRISM III score	0.82	0.78-0.86	<0.001	7.5	68%	82%
Blood lactate combined with PRISM III	0.86	0.83-0.90	<0.001		63%	93%
*P* value (comparison of the difference between AUCs)						
*p* = 0.318 (between blood lactate and PRISM III)						
*p* = 0.135 (between blood lactate combined with PRISM III and PRISM III alone)						
*p* = 0.018 (between blood lactate combined with PRISM III and blood lactate alone)						

*AUC*, area under the receiver-operating-characteristic curve; *CI*, confidence interval; *PRISM III,* pediatric risk of mortality III.

The sensitivity and specificity of the blood lactate levels and PRISM III scores at the optimal cut-off value to predict in-hospital mortality are shown in Table 4. Blood lactate displayed a sensitivity of 61% and a specificity of 86% at the optimal cut-off value of 5.55 mmol/l. The positive and negative likelihood ratios were 4.5 and 0.45, respectively. The PRISM III score displayed a sensitivity of 68% and a specificity of 82% for predicting in-hospital mortality at the optimal cut-off score of 7.5, and the positive and negative likelihood ratios were 3.8 and 0.39, respectively.

We also calculated the sensitivity and specificity of differing concentrations of blood lactate to predict in-hospital mortality in critically ill children (Table 5). At the cut-off value of >2.0 mmol/l, blood lactate displayed a sensitivity of 94% and a specificity of 24% for predicting in-hospital mortality, and the positive and negative likelihood ratios were 1.2 and 0.25, respectively. The specificity increased to 98%, and the positive likelihood ratio increased to 19.8 at the cut-off value of >10.0 mmol/l, although the sensitivity decreased to 34%. The OR values of blood lactate at the levels above the set cut-off points are shown in Table 5.

**Table 5:** Odds ratio, sensitivity, and specificity for admission blood lactate at different concentrations to predict in-hospital mortality in critically ill children

**Admission blood lactate mmom/L,**	**OR**^**a **^**(95% CI)**	***P *** **value**	**Sensitivity**	**Specificity**	**LR+**	**LR–**	**PV+**	**PV-**
>2.00	4.95 (2.27-10.78)	<0.001	94%	24%	1.2	0.25	0.55	0.80
>4.00	5.68 (3.72-8.68)	<0.001	70%	70%	2.4	0.42	0.70	0.70
>6.00	10.02 (6.59-15.23)	<0.001	55%	89%	5.1	0.51	0.84	0.66
>8.00	16.79 (10.37-27.19)	<0.001	43%	96%	10.1	0.60	0.91	0.63
>10.00	30.59 (16.55-56.53)	<0.001	34%	98%	19.8	0.67	0.95	0.60

*CI*, confidence interval; *LR+*, likelihood ratio positive; *LR-*, likelihood ratio negative; *OR*, odds ratio; *PV+*, positive predictive value*; PV-*, negative predictive value.

^a^Odds ratios of blood lactate at the levels above the set cut-off points.

In addition, 65.0% children had a lactate concentration >2.5 mmol/l in the study, which is suggested to be an optimal cut-off value for lactate to predict deterioration and mortality in adult patients [1]. Blood lactate displayed a sensitivity of 90% and a specificity of 38% to predict in-hospital mortality at this cut-off value in critically ill children.

## Discussion

This study provides data on blood lactate concentrations in critically ill children and demonstrates that the blood lactate level upon admission to a general medical PICU is significantly associated with in-hospital mortality. A high blood lactate level at PICU admission is predictive of in-hospital mortality in critically ill children.

Many studies have demonstrated that either admission lactate or peak lactate concentration is associated with mortality in adults [1,4,21,22]. To our knowledge, a limited number of studies verified the use of hyperlactatemia as a prognostic index in critically ill children who are admitted to the PICU [9,14,15]. Our results are in line with recently published findings that suggest that blood lactate concentration upon admission to PICU is predictive of mortality, independent of PIM2. Patients with contemporaneous blood lactate and PIM2 measurements at PICU admission were enrolled in a retrospective cohort study [14]. Our study was conducted in a large mixed cohort of critically ill children. Blood samples were prospectively collected for assessing the blood lactate concentration. The observation that the extent of absolute hyperlactatemia is strongly linked with mortality independent of illness severity indicates that blood lactate is a useful early predictor in identifying critically ill children who are at high risk of death in the pediatric intensive care setting.

The discrepancy between our data and the data from two studies performed on a small number of patients might most likely be attributed to the small sample size in the previous studies [9,15]. However, the possibility that the discrepancy might be caused by differences in the course of the clinical illness at PICU admission must be considered. Our study included a higher proportion of patients (859 of 1109, 77%) with a high level of blood lactate at admission (>2 mmol/l), compared to a previous publication in which high values were found in only 50 of the 705 studied children at admission (7%) [9]. Together with the high overall mortality of 10.6% in the present study, this finding implies that children might be admitted later to our PICU, most likely because of delayed PICU transfers.

One contribution of this study is the use of PRISM III to control for the severity of the illness. The PRISM III score is a valid measure of illness severity in the first 24 hours after admission and reflects the clinical picture of a child during the early admission period [19,23]. Previous studies suggest that PRISM III is an important tool in predicting mortality and clinical outcomes in the pediatric population [20,24,25]. The association of admission blood lactate with in-hospital mortality in this study was independent of age, gender, and the severity of illness as assessed by the PRISM III score. The ROC curve analysis in the present study showed that the prognostic accuracy of blood lactate for in-hospital mortality (AUC = 0.79) was similar to that of the PRISM III score (AUC = 0.82). Because blood lactate at admission and the PRISM III score obtained within the first 24 hours after PICU admission are comparable in predicting mortality, we recommend assessing mortality risk with blood lactate at admission because it is simple to use.

Previous studies suggest that there is a confounding relationship between hyperlactatemia and hyperglycemia in nondiabetic critically ill patients [26]. In the present study, there was a significant increase in the blood glucose concentration with an increase in the blood lactate levels, suggesting that hyperglycemia is significantly correlated with hyperlactatemia. We further demonstrated that hyperlactatemia was associated with an increased mortality risk, regardless of the presence of hyperglycemia. This finding suggests that hyperglycemia did not confound the association between the elevated blood lactate level and mortality in this study. In contrast, although hyperglycemia had a significant univariate association with mortality risk, when adjusted for concurrent hyperlactatemia and/or other potential risk factors, hyperglycemia was not a significant predictor of mortality risk in critically ill children.

The accumulation of lactic acid in the blood is generally associated with metabolic acidosis [12,27]. This finding raises the question of whether it is possible that the association of hyperlactatemia with mortality could be at least partially attributed to the occurrence of metabolic acidemia. We demonstrated that hyperlactatemia is significantly associated with in-hospital mortality in critically ill children, even after adjusting for arterial pH and blood bicarbonate.

This study was designed to determine the optimal relationship between the sensitivity and specificity of blood lactate assessment in predicting mortality and to set appropriate cut-off values for predicting in-hospital mortality. A systematic review conducted on studies of adult patients suggests that all patients with a lactate at admission above 2.5 mmol/l should be closely monitored for signs of deterioration [1]. In our study, 65.0% of the children had a lactate concentration >2.5 mmol/l. Blood lactate displayed a sensitivity of 90% and a specificity of 38% in predicting in-hospital mortality at 2.5 mmol/l. Specificity is one of the main characteristics of a predictive marker. A test with high sensitivity and low specificity carries the risk of many false positives. In the present study, an optimal cut-off for predicting in-hospital mortality in critically ill children appears to be a blood lactate level of 5.55 mmol/l, which has a sensitivity of 61% and a specificity of 86%. Our findings were similar to the results of a previous study conducted in children who were admitted to the PICU with a diagnosis of septic shock, where a lactate value of more than 5 mmol/l was an accurate predictor of death [28]. Our data, together with previous data, suggest that all critically ill children with high blood lactate at admission should be closely monitored for signs of clinical deterioration and children with low lactate levels should be considered for further monitoring of blood lactate, since serial lactate values may provide better prognostic information [1,6,9,29].

The limitations of the present study include a temporal mismatch in evaluating mortality indicators. We compared the prognostic performance of a lactate value obtained within 2 hours of admission to a PRISM III score, which considers a range of values and includes the worst values obtained in the first 24 hours of admission. The prognostic accuracy of the combination of blood lactate level and PRISM III score was not significantly better than the use of PRISM III alone (p = 0.135), which might be explained by the discrepancy in the timescales for these two methods. Notably, the primary aim of the study was to evaluate the predictive value of blood lactate, when measured as a screening method at admission, to predict mortality in critically ill children. The blood samples for lactate measurements were collected only within the first 2 hours of admission. Thus, serial changes in the lactate levels during the first 24 hours of admission were not evaluated. Trends in lactate concentration over time reflect the clinical response of patients to resuscitation. Serial measurements of lactate levels might improve the sensitivity and specificity of this prognostic test [29]. Further studies are needed to investigate the trends in the changes of lactate values and explore whether the addition of the highest lactate value in the first 24 hours to the PRISM III evaluation improves the prediction of mortality in the critically ill pediatric population.

A second limitation of this study is that the criteria for PICU admission vary widely. Our results might be biased by the fact that we conducted a single-center study. In our study, 77% of the children had an admission lactate level above 2 mmol/l, and 10.6% of the children died during the hospital stay. However, only 32% of children with an admission lactate level between 6 and 10 mmol/l had been mechanically ventilated. Our data imply that children might be admitted later to the PICU and received less aggressive treatment in our unit. These factors may limit the generalizability of our results to health care systems in which children are admitted earlier in their critical illness course and are managed more aggressively. A multicenter study is necessary to confirm our findings.

A third limitation of the study is that during the blood collection period, a substantial number of children received treatment with common PICU therapeutic interventions such as epinephrine. Epinephrine is known to affect lactate levels [30,31]. Unfortunately, we were unable to include these data in our multivariate analyses because we did not have information concerning therapeutic interventions for all children during their transfers to the PICU.

## Conclusions

Our study indicates that the blood lactate levels on admission to the PICU were significantly associated with mortality in critically ill children, even after adjusting for age, gender, and illness severity. A high level of blood lactate upon admission was independently predictive of in-hospital mortality in the pediatric population. These findings extend the knowledge of blood lactate as a clinical biomarker of mortality in critical illness.

## Abbreviations

AUC: Area under the receiver-operating-characteristic curve; CI: Confidence interval; MODS: Multi-organ dysfunction syndrome; OR: Odds ratio; PICU: Pediatric intensive care unit; PIM: Pediatric index of mortality; PRISM III: Pediatric risk of mortality III; ROC: Receiver operating characteristic.

## Competing interests

The authors declare that they have no competing interests.

## Authors’ contributions

ZJ Bai participated in study design and protocol development. XP Zhu carried out the data analysis and interpretation of data. MX Li and J Pan participated in clinical data collection. J Hua, Y Li, and J Wang participated in the design of the study and coordination. YH Li participated in data analysis, interpretation of data and writing of the manuscript. All authors read and approved the final manuscript.

## Pre-publication history

The pre-publication history for this paper can be accessed here:

http://www.biomedcentral.com/1471-2431/14/83/prepub

## References

[B1] Kruse O Grunnet N Barfod C Blood lactate as a predictor for in-hospital mortality in patients admitted acutely to hospital: a systematic review Scand J Trauma Resusc Emerg Med 2011 19 74 10.1186/1757-7241-19-74 22202128 PMC3292838

[B2] Kang YR Um SW Koh WJ Suh GY Chung MP Kim H Kwon OJ Jeon K Initial lactate level and mortality in septic shock patients with hepatic dysfunction Anaesth Intensive Care 2011 39 862 867 21970130 10.1177/0310057X1103900510

[B3] Nichol AD Egi M Pettila V Bellomo R French C Hart G Davies A Stachowski E Reade MC Bailey M Cooper DJ Relative hyperlactatemia and hospital mortality in critically ill patients: a retrospective multi-centre study Crit Care 2010 14 R25 10.1186/cc8888 20181242 PMC2875540

[B4] Martin J Blobner M Busch R Moser N Kochs E Luppa PB Point-of-care testing on admission to the intensive care unit: lactate and glucose independently predict mortality Clin Chem Lab Med 2013 51 405 412 22987833 10.1515/cclm-2012-0258

[B5] Jansen TC van Bommel J Bakker J Blood lactate monitoring in critically ill patients: a systematic health technology assessment Crit Care Med 2009 37 2827 2839 10.1097/CCM.0b013e3181a98899 19707124

[B6] Kalyanaraman M DeCampli WM Campbell AI Bhalala U Harmon TG Sandiford P McMahon CK Shore S Yeh TS Serial blood lactate levels as a predictor of mortality in children after cardiopulmonary bypass surgery Pediatr Crit Care Med 2008 9 285 288 10.1097/PCC.0b013e31816c6f31 18446112

[B7] Juneja D Singh O Dang R Admission hyperlactatemia: causes, incidence, and impact on outcome of patients admitted in a general medical intensive care unit J Crit Care 2011 26 316 320 10.1016/j.jcrc.2010.11.009 21255970

[B8] Ramakrishna B Graham SM Phiri A Mankhambo L Duke T Lactate as a predictor of mortality in Malawian children with WHO-defined pneumonia Arch Dis Child 2012 97 336 342 10.1136/archdischild-2011-300920 22267369

[B9] Hatherill M McIntyre AG Wattie M Murdoch IA Early hyperlactataemia in critically ill children Intensive Care Med 2000 26 314 318 10.1007/s001340051155 10823388

[B10] Duke T Butt W South M Karl TR Early markers of major adverse events in children after cardiac operations J Thorac Cardiovasc Surg 1997 114 1042 1052 10.1016/S0022-5223(97)70018-7 9434699

[B11] Siegel LB Dalton HJ Hertzog JH Hopkins RA Hannan RL Hauser GJ Initial postoperative serum lactate levels predict survival in children after open heart surgery Intensive Care Med 1996 22 1418 1423 10.1007/BF01709563 8986498

[B12] Hatherill M Waggie Z Purves L Reynolds L Argent A Mortality and the nature of metabolic acidosis in children with shock Intensive Care Med 2003 29 286 291 12594588 10.1007/s00134-002-1585-y

[B13] Duke TD Butt W South M Predictors of mortality and multiple organ failure in children with sepsis Intensive Care Med 1997 23 684 692 10.1007/s001340050394 9255650

[B14] Morris KP McShane P Stickley J Parslow RC The relationship between blood lactate concentration, the Paediatric Index of Mortality 2 (PIM2) and mortality in paediatric intensive care Intensive Care Med 2042–2046 2012 38 10.1007/s00134-012-2733-7 23100008

[B15] Koliski A Cat I Giraldi DJ Cat ML Blood lactate concentration as prognostic marker in critically ill children J Pediatr (Rio J) 2005 81 287 292 10.2223/JPED.1364 16106312

[B16] Cheifetz IM Kern FH Schulman SR Greeley WJ Ungerleider RM Meliones JN Serum lactates correlate with mortality after operations for complex congenital heart disease Ann Thorac Surg 1997 64 735 738 10.1016/S0003-4975(97)00527-4 9307466

[B17] American Academy of Pediatrics, Committee on Hospital Care and Section on Critical Care and Society of Critical Care Medicine, Pediatric Section Admission Criteria Task Force Guidelines for developing admission and discharge policies for the pediatric intensive care unit Pediatrics 1999 103 840 842 10103320

[B18] Goldstein B Giroir B Randolph A International pediatric sepsis consensus conference: definitions for sepsis and organ dysfunction in pediatrics Pediatr Crit Care Med 2005 6 2 8 10.1097/01.PCC.0000149131.72248.E6 15636651

[B19] Pollack MM Patel KM Ruttimann UE PRISM III: an updated pediatric risk of mortality score Crit Care Med 1996 24 743 752 10.1097/00003246-199605000-00004 8706448

[B20] Tan GH Tan TH Goh DY Yap HK Risk factors for predicting mortality in a paediatric intensive care unit Ann Acad Med Singapore 1998 27 813 818 10101556

[B21] Mikkelsen ME Miltiades AN Gaieski DF Goyal M Fuchs BD Shah CV Bellamy SL Christie JD Serum lactate is associated with mortality in severe sepsis independent of organ failure and shock Crit Care Med 2009 37 1670 1677 10.1097/CCM.0b013e31819fcf68 19325467

[B22] Gustavsson L Andersson LM Brink M Lindh M Westin J Venous lactate levels can be used to identify patients with poor outcome following community-onset norovirus enteritis Scand J Infect Dis 2012 44 782 787 10.3109/00365548.2012.686671 22831183

[B23] Wang JN Wu JM Chen YJ Validity of the updated pediatric risk of mortality score (PRISM III) in predicting the probability of mortality in a pediatric intensive care unit Acta Paediatr Taiwan 2001 42 333 337 11811220

[B24] Brady AR Harrison D Black S Jones S Rowan K Pearson G Ratcliffe J Parry GJ Assessment and optimization of mortality prediction tools for admissions to pediatric intensive care in the United kingdom Pediatrics 2006 117 e733 e742 10.1542/peds.2005-1853 16510615

[B25] Gemke RJ van Vught J Scoring systems in pediatric intensive care: PRISM III versus PIM Intensive Care Med 2002 28 204 207 10.1007/s00134-001-1185-2 11907665

[B26] Green JP Berger T Garg N Horeczko T Suarez A Radeos MS Hagar Y Panacek EA Hyperlactatemia affects the association of hyperglycemia with mortality in nondiabetic adults with sepsis Acad Emerg Med 2012 19 1268 1275 10.1111/acem.12015 23167858 PMC3506124

[B27] Gunnerson KJ Saul M He S Kellum JA Lactate versus non-lactate metabolic acidosis: a retrospective outcome evaluation of critically ill patients Crit Care 2006 10 R22 10.1186/cc3987 16507145 PMC1550830

[B28] Jat KR Jhamb U Gupta VK Serum lactate levels as the predictor of outcome in pediatric septic shock Indian J Crit Care Med 2011 15 102 107 10.4103/0972-5229.83017 21814374 PMC3145293

[B29] Charpie JR Dekeon MK Goldberg CS Mosca RS Bove EL Kulik TJ Serial blood lactate measurements predict early outcome after neonatal repair or palliation for complex congenital heart disease J Thorac Cardiovasc Surg 2000 120 73 80 10.1067/mtc.2000.106838 10884658

[B30] Day NP Phu NH Bethell DP Mai NT Chau TT Hien TT White NJ The effects of dopamine and adrenaline infusions on acid–base balance and systemic haemodynamics in severe infection Lancet 1996 348 219 223 10.1016/S0140-6736(96)09096-4 8684198

[B31] Levy B Bench-to-bedside review: is there a place for epinephrine in septic shock? Crit Care 2005 9 561 565 10.1186/cc3901 16356239 PMC1414043

